# Three decades of research in substance use disorder treatment for syringe services program participants: a scoping review of the literature

**DOI:** 10.1186/s13722-023-00394-x

**Published:** 2023-06-10

**Authors:** Andrea Jakubowski, Sabrina Fowler, Aaron D. Fox

**Affiliations:** 1 Department of Medicine, Division of General Internal Medicine, Albert Einstein College of Medicine/Montefiore Medical Center, 3300 Kossuth Avenue, Bronx, NY 10467 USA; 2 Present Address: Ascension St. John Hospital, 22101 Moross Road, Detroit, MI 48236 USA; 3 Albert Einstein College of Medicine, 1300 Morris Park Avenue, Bronx, NY 10461 USA

**Keywords:** Syringe services programs, Referral to treatment, People who inject drugs

## Abstract

**Background:**

Syringe services programs (SSPs) provide a spectrum of health services to people who use drugs, with many providing referral and linkage to substance use disorder (SUD) treatment, and some offering co-located treatment with medications for opioid use disorder (MOUD). The objective of this study was to review the evidence for SSPs as an entry point for SUD treatment with particular attention to co-located (onsite) MOUD.

**Methods:**

We performed a scoping review of the literature on SUD treatment for SSP participants. Our initial query in PubMed led to title and abstract screening of 3587 articles, followed by full text review of 173, leading to a final total of 51 relevant articles. Most articles fell into four categories: (1) description of SSP participants’ SUD treatment utilization; (2) interventions to link SSP participants to SUD treatment; (3) post-linkage SUD treatment outcomes; (4) onsite MOUD at SSPs.

**Results:**

SSP participation is associated with entering SUD treatment. Barriers to treatment entry for SSP participants include: use of stimulants, lack of health insurance, residing far from treatment programs, lack of available appointments, and work or childcare responsibilities. A small number of clinical trials demonstrate that two interventions (motivational enhancement therapy with financial incentives and strength-based case management) are effective for linking SSP participants to MOUD or any SUD treatment. SSP participants who initiate MOUD reduce their substance use, risk behaviors, and have moderate retention in treatment. An increasing number of SSPs across the United States offer onsite buprenorphine treatment, and a number of single-site studies demonstrate that patients who initiate buprenorphine treatment at SSPs reduce opioid use, risk behaviors, and have similar retention in treatment to patients in office-based treatment programs.

**Conclusions:**

SSPs can successfully refer participants to SUD treatment and deliver onsite buprenorphine treatment. Future studies should explore strategies to optimize the implementation of onsite buprenorphine. Because linkage rates were suboptimal for methadone, offering onsite methadone treatment at SSPs may be an appealing solution, but would require changes in federal regulations. In tandem with continuing to develop onsite treatment capacity, funding should support evidence-based linkage interventions and increasing accessibility, availability, affordability and acceptability of SUD treatment programs.

## Background

Syringe Services Programs (SSPs) are critical to the health of people who use drugs (PWUD), reducing HIV and Hepatitis C Virus (HCV) transmission through provision of counseling and sterile equipment for safer substance use [1, 2], offering overdose prevention services [3], facilitating access to other health and supportive services, and providing a “safe haven” for marginalized people [4]. In 2021, there were 370 known SSPs in 43 US states, Washington DC, and Puerto Rico that distributed 46 million syringes [5]. Though some US states still restrict SSPs and criminalize syringe possession as drug paraphernalia [6], many SSPs have expanded, adding additional services such as diagnostic testing for HIV or Hepatitis C Virus (HCV), case management services and even medical care [7, 8].

Working from a harm reduction framework, SSPs offer non-judgmental “low-barrier” care that often differs from care provided in conventional healthcare settings [9], where PWUD experience stigma from healthcare providers that deters them from seeking needed medical care [10]. SSPs reach a high-needs population. At least 80% of SSP participants report using heroin or other non-prescribed opioids, but use of stimulants, such as cocaine or methamphetamine, is also common [11]. Compared to other PWUD seeking treatment, SSP participants use non-prescribed opioids more frequently and are more likely to inject and share syringes [12]. One study of new SSP-users found a prevalence of HCV and HIV infection of 44.4% and 10.2%, respectively [13]. In 2019, approximately 29% of US SSPs offered primary care services and 50% offered wound care [8]. Because SSPs embrace a judgment-free approach to engaging PWUD [14], they may be optimal venues to provide medical services (referrals and care at the SSP), including substance use disorder (SUD) treatment; however, the effectiveness of medical interventions at SSPs has not been widely studied.

While SSPs undoubtedly provide health-promoting services to PWUD, their precise role in delivering evidence-based SUD treatment (medications for OUD (MOUDs) and behavioral treatments) deserves additional attention. A typical harm reduction framework places SUD treatment on a spectrum of person-centered goals regarding substance use, which can range from using in less risky ways (e.g., sniffing instead of injecting) to cutting down on substance use or even seeking abstinence [15]. While methadone, a first-line treatment for opioid use disorder (OUD), can only be administered in licensed opioid treatment programs, which are subject to state and federal regulations, buprenorphine, another first-line OUD treatment, can be offered in diverse office-based settings, including SSPs [16]. Behavioral interventions, such as motivational interviewing or cognitive behavioral therapy, could also be integrated into the services delivered at SSPs. Making a spectrum of effective services available at SSPs potentially could better meet the needs of PWUD than the current US healthcare system.

Our objective for this study was to review the evidence for SSPs as an entry point for SUD treatment, with particular attention to co-located MOUD. We found two prior reviews of SSP-based SUD treatment services. One focused exclusively on mobile SSPs described the variety of services provided in mobile settings and highlighted several studies demonstrating the success of referring mobile SSP participants to SUD treatment [17]. The second, conducted before MOUDs were commonly offered at SSPs, presented promising data on a small number of linkage-to-treatment interventions [18]. This scoping review aims to answer questions about the characteristics of SSP participants who enter SUD treatment, the effectiveness of interventions that link SSP participants to SUD treatment and outcomes after linkage, and the more recent expansion of co-located MOUD at SSPs.

## Methods

We conducted a scoping review in accordance with the Preferred Reporting Items of Systematic Reviews and Meta-Analyses for Scoping Review (PRISMA-ScR) checklist. A scoping review seeks to assess how a given topic is covered within a body of literature and is different than a systematic review, which synthesizes data in order to answer a more specific research question [19]. The study did not involve human subjects and therefore did not require IRB approval.

We consulted with a librarian at the D. Samuel Gottesman Library at the Albert Einstein College of Medicine to help finalize our search criteria and provide insight into the PRISMA-ScR guidelines. All researchers met to discuss the search criteria and finalize terminology for the keyword search. The initial search spanned referrals to SUD treatment, co-located buprenorphine or naltrexone prescribing, hereafter referred to as "onsite" treatment, linkage to other medical care, as well as HIV and HCV testing, treatment and prevention, and other medical services provided to SSP participants.

On February 21st, 2022 we performed a systematic keyword search (see Appendix A) on PubMed to assess the literature on medical and SUD services at SSPs. In short, our criteria required the presence of both “SSP” or similar term, plus mention of referrals/linkage, SUD treatment (including “detox” because SSPs facilitate linkage to medically supervised withdrawal at patient request), medical care, or MOUD. We did not include medications for other substance use disorders, as the majority of SSP participants use opioids [20]. No specific criteria were used for the date of publication, and the results spanned 1975 to 2022. Articles were filtered to include only those published in English. No additional filters were applied. We included studies from all countries to include innovative SUD treatment models that might be useful for clinicians and researchers in the United States, where federal regulations governing SUD treatment are changing to allow for more flexible treatment delivery. This search criteria yielded 3591 results, all of which were uploaded into a web-based platform (Covidence), which removed four duplicates. The remaining 3587 underwent title and abstract screening where two authors (SF and AJ) screened each title and abstract, determining whether the study fit inclusion criteria (yes, no or maybe). Studies met inclusion criteria if they were published in English and pertained to delivery of health services for SSP participants other than provision of sterile injection equipment. We excluded epidemiologic studies that examined population-level benefits of providing sterile injection equipment (i.e. reductions in HIV and HCV transmission). All articles receiving discrepant or maybe votes were identified as conflicts. SF and AJ then met to resolve these conflicts and make the final vote on these articles. At the end of this process, 3414 articles were excluded based on their title and abstract leaving 173 articles to be subjected to full-text review.

During the full-text review, the remaining articles were categorized by health service into: Medications for Opioid Use Disorder (MOUD) (N = 32), HIV Pre-Exposure Prophylaxis (PrEP) (N = 12), HCV testing or treatment (N = 41), HIV or HIV plus HCV testing and treatment (N = 22), motivational interviewing (N = 4), other (N = 31), or multiple services (N = 31). This review was also completed in Covidence with authors resolving conflicts in the same process used for the title and abstract screening. Given the large number of studies, we narrowed our scope to SUD treatment and excluded other health services from the final review, resulting in 46 studies. The focus of these 46 studies was: describing SSP participants’ SUD treatment utilization and entry (N = 11), onsite SUD treatment (N = 11), interventions to link SSP participants to SUD treatment (N = 8), post-linkage SUD treatment outcomes (N = 9), national surveys that describe health services (including SUD treatment) provided by SSPs (N = 2), SSP participants’ preferences regarding SUD treatment (N = 2), and review articles spanning categories (N = 3). Articles discussed in the three review articles were also reviewed for inclusion by a single researcher (AJ) resulting in an additional 12 articles being screened and two descriptive studies of SSP client’s SUD treatment utilization being added to this review. One of the two added studies did not have SSP or similar term in the title, which is likely why it was not identified in the initial search. We are uncertain why the second study was not identified in the initial search. Three additional articles on onsite SUD treatment published after the initial search date were also included based on the authors' knowledge of the field. Data were then extracted from the 51 included articles which spanned the years 1990–2022 and were conducted in six countries.

After full-text review, a data extraction template was created in Covidence (see Appendix B) and the 51 articles were divided amongst three authors for extraction. Following data extraction, all authors met to review findings and discuss conclusions that could be drawn from the body of literature. Studies that answered similar questions are presented together in the following sections with additional data presented in tabular format. Appendix C includes a full list of the 51 studies categorized by theme.

## Results

### What types of SUD treatment services do SSP participants want?

Two qualitative studies explored SSP participant's treatment preferences and attitudes toward MOUD options. Andraka-Christou et al. examined SSP participants’ preferences broadly for specialty SUD treatment centers and identified that participants wanted: (1) choice among multiple treatment modalities and levels of care (ranging from safe injection sites to sober living homes); (2) adjunctive social services such as classes, housing support and employment services; (3) family involvement; (4) integrated mental health treatment; (5) a harm reduction option; and (6) staff diversity, with a mix of formally trained professionals and recovery-experienced staff (e.g. peer support specialists) [21]. A study by Sohler et al. specifically examined SSP participants’ attitudes toward buprenorphine and methadone treatment recruiting from a single SSP in New York City [22]. Participants had more favorable attitudes toward buprenorphine than methadone, perceiving that buprenorphine would allow them greater control over their treatment, but participants also viewed methadone as a better option than buprenorphine for people who were not ready to fully abstain from non-prescribed opioids [22].

### Descriptive studies of treatment utilization/entry:

#### What characteristics of SSP participants are associated with SUD treatment utilization and entry?

We found 13 studies that examined substance use treatment entry and utilization among SSP participants (Table 1). Several of the studies examined patient characteristics associated with requesting and entering treatment as well as barriers to SUD treatment. We highlight client characteristics that were associated with treatment entry, which were found in one or more studies.

**Table 1:** Descriptive studies of treatment utilization/entry

First author and year, country (city/region)	Design	Sample description and N	Outcomes	Results
Altice, 2003, US (New Haven) [31]	Cohort study	HIV-positive PWID at an SSP (N = 13)	(a) Viral load and CD4 count; (b) entry into SUD treatment	(a) 85% had undetectable viral load at 6 months, 77% at 9 months and 54% at 12 months. Net increase in CD4 count = 150 cells/ml (b) 69% entered SUD treatment: 8 methadone,1 long-term residential program
Carvell, 1990, UK (London) [23]	Cross-sectional	SSP participants (N = 133)	Characteristics of SSP participants receiving and completing referrals to SUD treatment and medical services (n = 93) vs. not receiving referrals (n = 43)	Receiving and completing referrals vs. non-referred: mean age 17.3 vs. 18.9 (p < 0.05) age at 1st time injecting: 17.6 vs. 19.4 (p < 0.03) age daily injecting began: 18.8 vs. 21.2 (p < 0.003)
Deering, 2011, Canada (Vancouver) [30]	Cohort study	Female sex workers (N = 242)	(a) SUD treatment service utilization (b) Association between using peer-led mobile outreach program (including syringe services) (n = 97) vs. not using (n = 145) and SUD treatment utilization	(a) Of 479 observations over 18 months (N = 242): -Medically managed withdrawal: 7.5% -Residential SUD treatment: 4% -Methadone treatment: 28.8% -Outpatient SUD counseling: 9.6% (b) Using vs. not using the mobile program [AOR (95% CI)]:Used inpatient SUD treatment [4.2 (2.1–8.1)] Used outpatient SUD counseling [6.1 (2.6–14.2)]
Gervasoni, 2012, Switzerland [25]	Cross-sectional	SSP participants (N = 921)	Differences between those receiving methadone (n = 525) vs. not receiving (n = 386)	Receiving vs. not receiving methadone [AOR (95% CI)]: Female sex [1.6 (1.1–2.32)]; Age [1.38 (1.19–1.61)]; Lifetime injection [2.26 (1.52–3.36); HIV positive [2.69 (1.48–4.87)]; HCV positive [1.96 (1.49–2.60)]; Daily SSP visits [0.54 (0.41–0.72)]
Hagan, 2000, US (Seattle) [26]	Cohort study	PWID recruited from methadone and other SUD treatment programs, a correctional facility, and a street outreach and social service agency (N = 2879)	Differences between Ex-, Current, New, and Never SSP-users in: (a) Change in injection frequency (b) Entry into methadone treatment	(a) Reduced injection risk by 75% or more: Ex-SSP-users vs. Never SSP-users [ARR 2.85 (1.47–5.51)]; Ex-SSP users vs. Never SSP-users who inject daily or more [ARR 3.44 (1.46–8.09)] Stop injecting altogether: Ex-SSP users vs. Never SSP users (ARR 3.5, 95% CL 2.1–5.9)) (b) New vs. Never SSP users (ARR 5.05 [1.44–17.7)]
Heimer, 1998, US (New Haven) [28]	Cohort study	SSP participants (N = 1905)	(a) SSP participant characteristics associated with requesting treatment (all types) (n = 409) vs. not requesting treatment (n = 1496) (b) Factors associated with entering SUD treatment	(a) Treatment requesters vs. non-requesters: Female: 29.3% vs. 21.6% (p < 0.01) Race/ethnicity (p < 0.001) African American: 46.5% vs. 36.2% Latino: 26.7% vs. 20% White: 20.3% vs. 42.8% (b) 442 appointments made with 60% resulting in treatment entry. Barriers to entry include: treatment delays (< p = 0.01), no insurance (p < 0.01), cocaine use (p < 0.01)
Hudoba, 2004 Australia (New South Wales) [72]	Pre-Post	SSP workers (N = 6)	(a) Changes in referrals after staff training in counseling and harm reduction (b) Change in staff knowledge and confidence	(a) Referral rates (pre- to post-) for: Drug treatment: 44% to 49% (p < 0.01) Health and medical services: 23% to 28% (p < 0.01) (b) Changes (pre- to post-) in: Staff knowledge (p < 0.01) Confidence (p = 0.05)
Kidorf, 2010, US (Baltimore) [73]	Analysis of RCT data	Newly registered SSP participants who inject opioids (N = 281)	Association between psychiatric symptoms (global severity index (GSI) on the psychiatric Symptom Checklist-90) and treatment entry	High vs. low GSI scores predicted more treatment enrollment: AOR 2.15 (1.10–4.23)
Latkin, 2006, US (Baltimore) [27]	Cohort study	Out-of-treatment PWID (N = 440)	Characteristics associated with treatment entry (n = 166) vs. no treatment entry (n = 274)	Type of SUD treatment received: Medically managed withdrawal: 28.9%; Methadone treatment: 74.7%; Residential treatment: 22.3%; Outpatient program: 25.3% Characteristics associated with treatment entry: Recent SSP use [AOR 1.71, (1.12–2.62)]; Female sex [AOR: 1.77 (1.13–2.75)]; Recent intranasal cocaine use [AOR: 0.44, 0.21–0.91)]
Riley, 2002, US (Baltimore) [24]	1) Cohort study 2) Cross- Sectional study	1) SSP participants from a single SSP (N = requesting methadone treatment referral (N = 2659) 2) Survey respondents from 6 SSPs (N = 102)	1) Characteristics associated with requesting referral to SUD treatment (n = 139) vs. not requesting referral (n = 2520); Characteristics of referred patients who entered methadone treatment (n = 39) vs. did not enter methadone treatment (n = 100) 2) Patient reported barriers to entering treatment	1) AORs for requesting SUD treatment: Age > 38 [1.96 (1.63–2.37)]; Female sex [2.02 (1.63–2.37)]; Use speedballs in past 6 months [3.83 (3.14–4.66)]; > 20 years old at first injection [1.59 (1.31–1.94)] AORs for entering methadone treatment: Male [2.45 (1.04–5.78)] 2) Common barriers included: lack of health insurance, lack of treatment slots, residing too far from treatment facility, employment schedule conflicts, incarceration, expectation of overwhelming or difficult paperwork
Strathdee, 1999, US (Baltimore) [29]	Cohort study	PWID (N = 1483)	1) Predictors of entry into medically managed withdrawal by HIV status, including SSP attendance	AORs for entry into medically managed withdrawal (among HIV negative and HIV positive): Injecting > once/day [2.03 (1.48–2.78) and 2.98 (1.66–5.35)]; Injecting speedballs or heroin [2.22 (1.3–3.77)] (HIV positive only); Admitted to hospital [4.0 (3.08–5.19) and 2.82 (1.28–4.38)]; Visited physician [1.92 (1.28–2.89)] (HIV positive only); Health insurance [1.51 (1.01–2.28)] (HIV positive only); Attended SSP [1.38 (1.02–1.87) and 3.2 (1.38–7.53)]
Surratt, 2020 US (Appalachian Kentucky) [32]	Cross-sectional	PWID from 3 SSPs (N = 186)	Characteristics of SSP participants associated with current treatment participation	Uptake of the SSP: 44.6% had 6 + visits over the past 6 months AORs for current treatment participation: 6 + SSP visits/6 months [1.368 (0.557–3.362)] (NS); Confident in reducing substance use [2.241 (1.002–5.009)]; Primary injection drug methamphetamine [0.341 (0.146–0.798)]

*SSP* syringe services program, *PWID* people who inject drugs, *SUD* substance use disorder, *GSI* global severity index

*Age:* Studies were mixed on the association between age and treatment entry. In a UK cross-sectional study (N = 133), SSP participants with earlier age of onset of opioid use, injection use and daily injection drug use were significantly more likely to be referred to and accept SUD treatment, though no significant differences in participant age at the time of referral were noted [23]. This contrasts with other studies showing that older age was associated with SSP participants requesting and receiving methadone treatment [24, 25].

*Sex*: Findings on the association between sex and treatment entry were mixed. Four studies found that female SSP participants were significantly more likely to be receiving methadone treatment [25], request SUD treatment [24, 26], and enter SUD treatment [27] than males. However, one study found that males were significantly more likely to enter SUD treatment than women [24]. A possible confounder of sex differences seen in SSP participants regarding SUD treatment entry is living with children, which was found to be both a significant barrier to requesting methadone treatment and entering methadone treatment in one study [24].

*Race/ethnicity:* The only significant difference by race/ethnicity that we found were described in a US study conducted at a single urban SSP (N = 1905), where Black SSP participants and Latino SSP participants were more likely to request SUD treatment (for any substance, inpatient or outpatient setting, MOUD and medically-managed withdrawal for OUD) than white SSP participants [28].

*Stimulant use:* Data were mixed on whether stimulant use was associated with SUD treatment entry. History of using speedballs (i.e., cocaine and heroin concomitantly) was significantly associated with higher likelihood of requesting methadone treatment in one study [24], and higher odds of entering medically managed withdrawal (i.e., detoxification) among HIV-positive people who inject drugs (PWID) [29]; however, cocaine use was associated with significantly lower likelihood of entry into methadone treatment in one study [26], and methadone or other SUD treatment in another study [27].

#### Is SSP participation associated with entry into SUD treatment?

Involvement in SSPs was found to help facilitate entry into treatment. Several studies of PWID demonstrated that SSP participants were significantly more likely than non-SSP participants to enter methadone treatment, medically managed withdrawal, or other SUD treatment, and to stop injecting [26, 27, 29, 30]. A small pilot that provided HIV treatment at a mobile SSP found that 9 of 13 participants subsequently entered SUD treatment (8 methadone and 1 residential), indicating that co-locating medical services with SSPs may promote treatment entry [31]. We found two studies with conflicting results about the association between frequency of attending SSPs and entering SUD treatment. A Swiss cross-sectional study of 921 SSP participants found that participants who attended SSPs daily were less likely to be currently receiving methadone treatment than those who visited less frequently than daily [25]. By contrast, a cross-sectional study of 186 SSP participants conducted in Kentucky found that more frequent SSP visits (greater than monthly) was non-significantly associated with greater odds of participation in any SUD treatment and significantly associated with greater confidence in reducing substance use [32].

#### What barriers do SSP participants face in accessing SUD treatment?

There are numerous barriers to linking SSP participants to SUD treatment. A cross-sectional study with 102 SSP participants found barriers to SUD treatment entry included: lack of health insurance, unavailable treatment slots, residing too far from treatment facilities, work conflicts, incarceration, and expecting overwhelming or difficult paperwork [24]. Many SSP participants also are unaware of the availability of referrals, onsite treatment, and other medical services at SSPs [24, 33], including referrals to SUD treatment. One study found that among SSP participants who used non-prescription buprenorphine (56% of the sample), the majority did not know where to go for formal buprenorphine treatment [34].

### Linkage trials: what strategies effectively link SSP participants to SUD treatment?

We found three US randomized controlled trials (RCTs) that examined strategies to link SSP participants to SUD treatment (MOUD, inpatient and outpatient medically-managed withdrawal, and “drug-free” modalities) (Table 2) [35–37]. In these three trials, the proportion of participants successfully linked to methadone was 9–26% in control conditions and 8–40% in linkage conditions. In Kidorf 2005, a single 50-min motivational interviewing intervention was compared with two control groups (an attention control and standard referral) [36]. The intervention was found to be no better than control conditions for linkage to any SUD treatment or methadone treatment specifically. In Kidorf 2009, an enhanced motivational referral condition (MRC), which included 8 one-hour motivational enhancement sessions plus 16 one-hour treatment readiness groups, was compared to MRC with financial incentives or a standard referral condition for linkage to any treatment and methadone treatment [35]. Only MRC with financial incentives was superior to referral alone. In follow-up studies that reported longer term outcomes and SUD treatment *re-*enrollment after stopping treatment, MRC with financial incentives maintained superiority to MRC alone and standard referral [38, 39]. In Strathdee 2006, strength-based case management (SBCM), consisting of rapport building, strengths assessment, goal-setting, and linkage to a variety of support services (duration and frequency of case management was participant-driven), was compared to passive referral for linkage to methadone or levo-alpha acetyl methadol (LAAM) [37]. SBCM was superior to referral for linkage to methadone or LAAM. Receiving more case management services was significantly associated with successful linkage in adjusted logistic regression models [37]. Outside of the US, in a small Swedish study, Bråbäck et al. compared a case management intervention to referral alone for linkage to methadone and buprenorphine treatment for SSP participants (N = 75) [40]. Treatment entry rates were much higher than in US studies (95% for case management vs. 94% in the control condition), likely reflecting regional differences in treatment delivery. Finally, in a small single arm trial where SSP staff were trained to link participants to a clinic that provided buprenorphine treatment, there were no differences in linkage pre and post-intervention; however, the “dose” of staff training was not described [41]. In sum, motivational interviewing when combined with financial incentives, and strength based case management are evidence-based interventions to increase linkage to MOUD and other SUD treatment.

**Table 2:** Clinical trials of linkage interventions

First author and year, country (city)	Conditions	Sample description and N	Outcomes	Results
Bråbäck 2016, Sweden (Malmo) [40]	Intervention arm: Case management plus referral Comparison arm: Referral alone	People with OUD enrolled in a single SSP (N = 75)	% entering methadone or buprenorphine treatment	95% Case management intervention vs. 94% control (NS)
Kidorf 2005, USA (Baltimore) [36]	Intervention arm: Motivational Interviewing plus referral Comparison arms: 1) Attention control (job readiness (JR) training) 2) Standard Referral (SR)	People with OUD newly registering in a single SSP (n = 302)	% enrolling in (a) any treatment enrollment or (b) methadone treatment	(a) Any treatment: MI vs. JR vs. SR (10% vs. 13% vs. 10%, NS) (b) Methadone: MI vs. JR vs. SR (8% vs. 10% vs. 9%, NS)
Kidorf 2009, USA (Baltimore) [35]	Intervention arms: 1) Motivational referral condition (MRC) 2) MRC + financial incentives (MRC + I) Comparison arm: Standard referral condition (SRC)	People with OUD newly registering in a single SSP (n = 281)	% enrolling in (a) any treatment or (b) methadone treatment	(a) Any treatment: MRC + I vs. MRC vs. SRC (52.1% vs. 31.9% vs. 35.5%; p = 0.01) (b) Methadone: MRC + I vs. MRC vs. SRC (40.4% vs. 20.2% vs. 16.1%; p < 0.01)
Kidorf 2011, USA (Baltimore) [38]	*Same RCT as Kidorf 2009* Intervention arms: 1) Motivational referral condition (MRC) 2) MRC + financial incentives (MRC + I) Comparison arm: Standard referral condition (SRC)	RCT participants from Kidorf 2009 who enrolled in any SUD treatment within 4 months of SSP enrollment (N = 113)	**%** restarting (a) any treatment or (b) methadone treatment after stopping treatment	(a) Any treatment: MRC + I vs. MRC vs. SRC (64.4% vs. 28.0% vs. 37.0%; p < 0.01) (b) Methadone: MRC + I vs. MRC vs. SRC (44.4% vs. 12.0% vs. 3.7% SRC; p < 0.01)
Kidorf 2012, USA (Baltimore) [39]	*Same RCT at Kidorf 2009* Intervention arms: 1) Motivational referral condition (MRC) 2) MRC + financial incentives (MRC + I) Comparison arm: Standard referral condition (SRC)	RCT participants from Kidorf 2009 (N = 281)	% enrolling in (a) any treatment or (b) methadone treatment enrollment; (c) days of heroin use per month at 12 months	(a) Any treatment: MRC + I vs. MRC vs. SRC (62.8% vs 52.1% vs. 50.5%, NS) (b) Methadone: MRC + I vs. MRC vs. SRC (46.8% vs. 24.7% vs. 26.6%; p < 0.01) (c) Heroin use days: MRC + I vs. MRC vs. SRC (18.1 (0.84) vs. 24.1 (0.85) vs. 23.5 (0.88); p < 0.01)
Strathdee 2006, USA (Baltimore**) **[37]	Intervention arm: Strength based case management (SBCM) Comparison arm: passive referral	SSP participants who sought SUD treatment (N = 245)	% enrolling in treatment (methadone or levo-alpha-acetyl-methadol (LAAM)) within 7 days of referral	40% in SBCM vs. 26% of passive referral (p = 0.03)
Havens 2007, USA, (Baltimore) [44]	*Same RCT as Strathdee 2006* Intervention arm: Strength based case management (SBCM) Comparison arm: passive referral	RCT participants from Strathdee 2006 who completed 1 month follow up visit (N = 162)	Association of anti-social personality disorder (ASPD) with treatment entry (methadone or levo-alpha-acetyl-methadol (LAAM)) within 7 days of referral	AOR for treatment entry among participants with ASPD: 3.51 (1.04–11.9)
Fox 2017, USA (NYC) [41]	Single arm trial: single SSP received intensive multi-component staff training in buprenorphine linkage	N = 76 SSP participants N = 22 SSP staff members trained	% enrolling in buprenorphine treatment pre- and post-intervention	Pre-intervention: 4% vs Post-intervention: 0%, NS

*SSP* syringe services program, *MOUD* medication for opioid use disorder, *OUD* opioid use disorder, *SUD* substance use disorder

### Post-linkage outcomes: what are SUD treatment outcomes among SSP participants?

Studies fell into two categories: (1) assessing long-term outcomes (primarily SUD treatment retention) of SSP participants who were linked to treatment; and (2) comparing SUD treatment outcomes between patients referred from SSPs versus patients referred from other sources (Table 3).

**Table 3:** Post-linkage outcomes

First author and year, country (city)	Design	Sample description and N	Outcomes	Results
Havens 2009, USA, (Baltimore) [74]	*Same RCT as Strathdee 2006* Intervention arm: Strength based case management (SBCM) Comparison arm: passive referral	RCT participants who successfully entered treatment (N = 127)	Predictors of retention in methadone treatment	Median duration in treatment was 7.9 months (IQR = 6–12.2 months) Adjusted HR for days retained in methadone: Treatment site ≥ 4.5 miles from home 2.15 (1.31–3.51); Lived in more than one place in last year 1.79 (1.10–2.92); Unemployed 0.37 (0.21–0.63); Previously enrolled in outpatient drug-free treatment 0.3 (0.13–0.71); Asked SSP twice or more for treatment slot 0.60 (0.36–0.97); Baseline ASI psychiatric score ≥ median score 2.22 (1.33–3.73)
Bråbäck 2017, Sweden (Malmo)[43]	*Same RCT as Bråbäck 2016* Intervention arm: CASE management plus referral Comparison arm: Referral alone	RCT participants who successfully enrolled in buprenorphine or methadone treatment (N = 71)	Treatment retention	3, 6 and 12 month retention: 94%, 89%, 82%
Kidorf 2018, USA (Baltimore) [42]	Intervention arms: 1) Low Threshold methadone Initiation (LTI) 2) Voucher Reinforcement Initiation (VRI) Comparison arm: Standard Care Initiation (SCI)	SSP participants newly initiating methadone treatment (N = 212)	Treatment retention	3 month retention: (SCI: 31%; VRI: 34%; LTI: 35%, NS) 6 month retention: (SCI: 29%; VRI: 34%; LTI: 37%, NS)
Kidorf 2021, United States (Baltimore) [46]	*Same RCT as Kidorf 2018* Intervention arms: 1) Low Threshold methadone Initiation (LTI) 2) Voucher Reinforcement Initiation (VRI) Comparison arm: Standard Care Initiation (SCI)	RCT participants (N = 210)	Sexual risk behaviors over 6 months after starting methadone treatment	Sexual risk scores (range 0–18) decreased by 0.354 one month after enrollment (p < 0.01), but not significantly thereafter (p = 0.321)
Kidorf 2021, United States (Baltimore) [45]	*Same RCT as Kidorf 2018* Intervention arms: 1) Low Threshold methadone Initiation (LTI) 2) Voucher Reinforcement Initiation (VRI) Comparison arm: Standard Care Initiation (SCI)	RCT participants (N = 210)	Substance use risk behaviors over 6 months after starting methadone treatment	Drug risk scores (range: 0–22) decreased by 1.573 one month after enrollment (p < 0.01), with reductions of 0.129 per month thereafter (p < 0.01)
Kidorf 2011, United States (Baltimore) [75]	*Same RCT as Kidorf 2009* Intervention arms: 1) Motivational referral condition (MRC) 2) MRC + financial incentives (MRC + I) Comparison arm: Standard referral condition (SRC)	RCT participants who enrolled in methadone treatment vs. did not enroll (N = 240)	Risk behaviors over 4 months	Participants who enrolled in treatment reported less days of opioid (p < 0.01) and cocaine (p < 0.01) use, IVDU (p < 0.01), illegal activities (p < 0.01) and incarceration (p < 0.05) after 4 months
Kuo 2003, United States (Baltimore) [76]	Cohort study	People with OUD enrolled in a single SSP for > 1 month who expressed interest in SUD treatment (N = 163)	(a) % enrolling in treatment (levomethadyl acetate hydrochloride (LAAM)), (b) treatment retention, and (c) early treatment response (UDT) and addiction severity index (ASI) scores	(a) 70% enrolled in LAAM (b) 84% actively enrolled after 90 days, mean duration treatment 8.1 months (c) 31.2% decrease in heroin positive (p < 0.01) and 22.5% decrease in cocaine positive (p = 0.01) UDT from month 1 to 3. ASI scores lower in drug (p < 0.01), alcohol (p < 0.01) and legal (p < 0.01) domain at 1 month
Brooner 1998, United States (Baltimore) [12]	Cohort study	Patients admitted to methadone treatment referred from SSP vs. standard referral sources (N = 325)	(a) Treatment retention & (b) substance use	(a) 76% of SSP referred vs. 88% of other completed 13 weeks of treatment (p > 0.01); (b) SSP referred had more opioid positive (49% vs. 29% p < 0.01) and cocaine positive (54% vs. 32% p < 0.01) than standard referral sources during first 3 months of treatment
Neufeld 2008, United States (Baltimore) [47]	Same study as Brooner 1998 Cohort study, outcomes extended to 12 months	Patients admitted to methadone treatment referred from SSP vs. other referral sources (N = 324)	(a) Treatment retention at 12 months and (b) substance use (UDT)	(a) 35% SSP referrals vs. 56% other referral source completed 365 days of treatment (p < 0.05); (b) SSP referred had negative UDTs for opioids (63% vs. 78%, p < 0.05), cocaine (57% vs. 75%, p < 0.05) and any drugs (45% vs. 62%, p < 0.05)

*SSP* syringe services program, *MOUD* medication for opioid use disorder, *OUD* opioid use disorder, *SUD* substance use disorder, *ASI* addiction severity index, *HR* hazards ratio, *UDT* urine drug testing

#### Long-term SUD treatment outcomes

For the first category, 90-day retention in methadone or buprenorphine treatment for SSP participants was less than one-third in one US study [42] and 94% in a Swedish study [43]. In a follow-up study of Strathdee et al.’s SBCM intervention (which was effective for linkage) [37], once participants were linked to MOUD, retention was high (69% retained for 90 days or more) [44]. In Kidorf 2018, participants were randomized to one of three strategies to help SSP participants who were newly initiating methadone treatment (Low-threshold treatment, which required minimal counseling and allowed flexible dosing times; Voucher reinforcement for adhering to scheduled dosing and counseling sessions (ranging from $12 per week initially to max of $174 per week); and Standard care) [42]. There were no significant differences in retention between the three groups at 90 and 180 days [42]. Subsequent analyses of associations between treatment retention and risk behaviors showed that those retained in treatment also reduced non-prescription opioid use, drug risk behaviors, and sexual risk behaviors, such as number of partners, frequency of transactional sex, and frequency of condom use [45, 46].

#### Comparing outcomes between SSP participants and other patients

For the second category, two studies compared outcomes between SSP participants and non-SSP participants who entered methadone treatment. Compared to non-SSP participants, SSP participants had higher scores on the addiction severity index (ASI) and greater substance use at baseline [12]. While SSP participants were less likely than non-SSP participants to be retained in methadone treatment or abstain from heroin, cocaine and other drugs while in treatment, 76% of those referred from SSPs were retained for 90 days or more [12] and 35% for one year or more [47].

In conclusion, many SSP participants who initiate MOUD are retained in treatment and reduce their substance use and risk behaviors, though they may be less likely to achieve abstinence than non-SSP participants.

### Onsite MOUD at SSPs

#### Do SSP participants want to receive MOUD onsite at SSPs?

Quantitative and qualitative studies have explored SSP participants’ attitudes toward onsite SUD treatment. Fox et al. surveyed 102 SSP participants who used opioids to assess preferences for SUD treatment, finding that most participants (51%) would prefer to receive buprenorphine treatment onsite at SSPs as opposed to referral to specialty SUD treatment or general medical clinics [48]. A later cross-sectional study of SSP participants’ preferences for what formulation of buprenorphine they would like to receive, showed that of SSP participants who were receiving or considering buprenorphine treatment, most would prefer the sublingual formulation (50%), while 38% would prefer the injectable formulation (due to the convenience of a monthly injection) [49].

Two qualitative studies also examined SSP participants’ attitudes toward onsite treatment. In Sohler 2013, participants reported that buprenorphine treatment was not accessible in their communities, but they had mixed opinions about providing buprenorphine onsite in SSPs as opposed to traditional clinic settings [22]. Some participants reported they would feel more comfortable getting treatment onsite at the SSP, while other participants thought onsite treatment would be "too easy" and engage people who were not ready to abstain from drugs. Another qualitative study specifically probed SSP participants’ attitudes toward onsite buprenorphine treatment at SSPs [50]. Participants contrasted the non-judgmental environment and trusting relationships they had with the SSP to the stigma and negative experiences they had experienced in traditional SUD treatment settings. They also expressed concerns that onsite treatment could change the SSP’s culture by bringing in participants wanting treatment not harm reduction interventions, “institutionalizing” the SSP, and making the SSP more “sterile” and like a doctor’s office. Finally, participants voiced concerns that buprenorphine diversion, while driven by lack of access to treatment, could put the SSP at risk of being shut down. However, participants believed this risk could be reduced in a well-run program [50]. The majority of participants also recommended separate waiting areas for SSP participants receiving harm reduction services and those receiving buprenorphine treatment to minimize triggers for those striving for abstinence [50].

Thus, SSP participants are interested in onsite buprenorphine treatment (sublingual and long-acting injectable buprenorphine) at SSPs, which are trusted community resources. Unique considerations to offering onsite treatment include the harm-reduction ethos, avoiding over-medicalization, balancing concerns about medication diversion with the urgency of expanding access, and acknowledging that some SSP participants will not want buprenorphine either onsite or in other settings.

#### Are SSPs currently offering onsite MOUD?

Where historically SSPs primarily made referrals to SUD treatment, there are now many SSPs that provide buprenorphine treatment in the US. A cross-sectional study of 153 SSPs nationwide examined changes in services prior to the COVID-19 pandemic (2019) and during the COVID-19 pandemic (2020) [8]. The percentage of programs in 2019 that provided onsite buprenorphine, naltrexone and methadone treatment was 19.9%, 12.3% and 3.4%, respectively, and did not change significantly in 2020. The proportion of SSPs providing MOUD telehealth services increased from 3% in 2019 to 8% in 2020 [8]. A qualitative study of buprenorphine services implementation in 8 NYC SSPs identified key implementation barriers and facilitators [51]. Barriers included gaps in staff knowledge and comfort communicating with participants about buprenorphine, difficulty hiring buprenorphine providers, managing tension between harm reduction and traditional OUD treatment philosophies, and financial constraints. Facilitators included technical assistance from the city public health department, designating SSP staff as buprenorphine coordinators (responsible for patient navigation, communicating with providers, and tracking patients) offering other supportive services to participants, and using telehealth to bridge gaps in provider availability [51]. The COVID-19 pandemic also provided an opportunity for SSPs to expand buprenorphine access through telehealth with the 2020 waiver of the Ryan Haight Act, which mandates in-person visits for controlled substances-prescribing. In a 2020 national survey of US SSPs, 24% reported offering buprenorphine initiation via telehealth [52]. Characteristics of SSPs associated with offering telehealth buprenorphine initiation included being a non-governmental SSPs (vs. governmental SSPs), having a larger budget, and being located in the Northeast [52]. A study of an onsite buprenorphine telehealth program at SSPs across California found that of the 115 SSP participants served, 87% initiated buprenorphine the same day they were referred and 64% returned for a second buprenorphine prescription.[53] In summary, increasing numbers of SSPs are overcoming barriers to implement onsite buprenorphine services. Telehealth is a promising way to expand onsite buprenorphine treatment.

#### What are the outcomes when SSP participants receive onsite MOUD?

We found seven studies that reported onsite MOUD outcomes (Table 4). These papers examined buprenorphine treatment retention and changes in non-prescribed opioid use. The proportion of patients retained at three months ranged from 27 to 77% (N = 4 studies), six months: 31–65% (N = 4 studies), and 12 months: 20–59% (N = 3 studies). These buprenorphine treatment retention rates are similar to those reported in traditional office-based buprenorphine treatment programs [15]. SSP participants also reduced their opioid use while in treatment, though there was variability in how this was reported [54–58]. One study found that 79% of participants had one or more opioid-positive urine drug test (UDT) at 12 months [59], while another study found 16% with an opioid-positive UDT at 12 months [57]. Importantly, one study that reported only small reductions in opioid use based on UDTs, also identified reductions in HIV risk behaviors (drug and sexual risk behaviors) and opioid overdose among those retained in treatment [58]. Thus, SSP participants are retained in buprenorphine at rates that are comparable to other office-based settings and many reduce their opioid use, overdose risk, and HIV risk behaviors while in treatment.

**Table 4:** Onsite buprenorphine treatment at syringe services programs

First author and year, country (city)	Design	Sample description and N	Outcomes	Results
Bachhuber 2018, USA (Philadelphia) [77]	Cohort study	SSP participants who initiated onsite buprenorphine treatment (N = 124)	(a) Treatment Retention; (b) % participants with positive buprenorphine and opioid UDT	(a) 3, 6, 9 and 12-month retention: 77%, 65%, 59%, 56%; (b) Positive buprenorphine UDT at 3, 6, 9, and 12 months: 88% (n = 50/57), 100% (n = 31/31), 96% (n = 23/24), and 95% (n = 18/19); positive opioid UDT at 3, 6, 9, and 12 months were 19% (n = 11/57),13% (n = 4/31), 17% (n = 4/24), and 16% (n = 3/19)
Hill 2022, USA (D.C.) [55]	Cohort study	SSP participants who initiated onsite HCV treatment and buprenorphine treatment (N = 49)	(a) Treatment Retention (buprenorphine treatment); (b) % participants with positive buprenorphine urine drug test (UDT) and opioid UDT	(a) 1, 6 and 12-month retention: 82%, 65%, 59%; (b) Positive buprenorphine UDT at 1, 6 and 12 months: 80% (n = 32/40), 88% (n = 28/32), 79% (n = 23/29); (c) opioid positive UDT at 1, 6 and 12 months: 73% (n = 29/40, 56% (n = 18/32), 79% (n = 23/29)
Hood 2020, USA (Seattle) [54]	Cohort study	SSP participants who initiated onsite buprenorphine treatment (N = 146)	(a) Treatment Retention (time in treatment, median days); (b) % urine drug tests (UDT) positive for buprenorphine and opioid UDT	(a) Retention: 21 days (IQR 5–154) for patients with a single care episode, 76 days (IQR 24–129) for patients with intermittent care; (b) Between the first and sixth visits, % positive buprenorphine UDTs increased (33% to 96%, P < 0.01) and other opioids decreased (90% to 41%, P < 0.01)
Jakubowski 2022, USA (New York City) [78]	Cohort study	SSP participants who initiated onsite buprenorphine treatment (N = 118)	(a) Treatment Retention; (b) Patient characteristics associated with retention; (c) Buprenorphine and illicit opioid use by UDT (of 82 participants with 2 or more UDT)	(a) 1, 3 and 6 month retention: 62%, 43%, 31%; (b) Retained vs. not retained at 180 days: Current IDU: 42% vs. 66% (p < 0.01); (c) Median percentage of buprenorphine-positive UDTs was 71% (IQR 40–100%) and opioid-positive UDTs was 50% (IQR 11–100%)
Rosecrans 2022, USA (Baltimore) [79]	Cohort study	Participants who initiate buprenorphine from mobile vans that offer buprenorphine and other medical services (N = 420)	(a) Treatment Retention; (b) Factors associated with retention	(a) 1 and 3-month retention: 56.0%, 26.9%; (b) Factors associated with 3-month retention: white race (vs. Black race): (AOR = 0.32, 95% CI 0.16–0.61); Receiving HCV treatment: AOR = 5.51, 95% CI 1.99–15.27)
Rosenthal 2020, USA (D.C.) [58]	Single arm trial	SSP participants with HCV, OUD, and injection drug use (N = 100)	(a) Uptake buprenorphine treatment; (b) Treatment Retention; (c) % urine drug tests (UDTs) positive for opioids	(a) Thirty-three (33%) patients were receiving MOUD at screening Of the 67 not receiving MOUD, 53 (79%) started buprenorphine treatment; (b) At week 24, 68 (68%) patients were receiving MOUD; (c) opioid-positive UDTs: baseline (80%), 4 weeks (77%), 12 weeks (70%) and 24 weeks (68%)
Stancliff 2012, USA (New York City) [80]	Cohort study	SSP participants who initiated buprenorphine treatment (N = 100)	(a) Treatment retention; (b) Factors associated with retention	(a) 3, 6, 9 and 12-month retention: 42%, 31%, 28%, 20%; (b) Factors associated with active prescriptions: African American race 41% vs. 21%; Latino 29% vs. 50%

*SSP* syringe services program, *MOUD* medication for opioid use disorder, *OUD* opioid use disorder, *SUD* substance use disorder, *UDT* urine drug testing, *IDU* injection drug use

## Conclusions

Over 20 years ago, clinical researchers asked whether syringe services programs could serve as a “conduit” to substance use disorder treatment [28]. Our scoping review reveals observational research and clinical trials that answer this question. Studies demonstrate that SSP participants are interested in SUD treatment, and while passive referral leads to suboptimal linkage rates, motivational enhancement plus financial incentives or strengths-based case management is likely more effective. SSP participants who successfully link to SUD treatment benefit clinically, but outcomes, such as treatment retention, may be somewhat lower than those for persons referred to treatment from other sources. Nonetheless, studies also demonstrated that SSP participants would prefer to receive evidence-based SUD treatment directly onsite at SSPs and several clinical programs have published promising results. Therefore, SSPs can facilitate SUD treatment, but there are clear opportunities to improve SUD treatment delivery for their participants.

Prior studies have demonstrated that PWID who use SSPs have higher injection risk behaviors and more frequent overdoses than other PWID who do not use SSPs or use them less often [60, 61]. Our review reinforces some of this selection effect. There is an extensive literature on MOUD outcomes, and the retention rates and reductions in non-prescribed opioid use reported for SSP participants in the studies we reviewed were somewhat lower than those seen in prior clinical trials and observational studies [62, 63]. SSP participants frequently use multiple substances (e.g. opioids, stimulants, alcohol or benzodiazepines), and of SSP participant characteristics most strongly associated with entering SUD treatment, using stimulants was negatively associated with SUD treatment entry. Lack of health insurance and transportation were also identified as barriers to SUD treatment. Therefore, SSPs likely are successful in engaging persons who could benefit from SUD treatment, but additional supports, which could be integrated into SSPs with sufficient funding, will likely be necessary to facilitate starting treatment and maintaining treatment retention.

Our finding that passive referral to SUD treatment was challenging in US-based studies is not surprising. Challenges with linkage to specialty services are not unique to SUDs; referral to specialty HCV and mental health services is also suboptimal, even from traditional primary care settings [64, 65]. While motivational enhancement with financial incentives and strengths-based case management boosted referral rates, the results of this review demonstrate that more robust interventions are needed. Waiting to enter SUD treatment—for example, over a weekend when programs are closed—may be intolerable for persons who are experiencing withdrawal from opioids or other substances. Our research group is testing a strategy of facilitating referral to buprenorphine treatment with a bridging prescription that is offered onsite at SSPs [66]. This approach of initiating buprenorphine treatment and facilitating referral has been successful in the emergency department and general hospital settings [67, 68]. The Drug Enforcement Administration has also recently clarified the “72 h rule,” which allows controlled substances such as methadone and buprenorphine to be administered in general medical settings for 3 consecutive days to treat withdrawal symptoms and facilitate treatment initiation [69]. The feasibility of storing and administering methadone and buprenorphine in an SSP deserves additional attention. Other strategies for facilitating referral could include peer navigation, transportation vouchers, and decreasing barriers to treatment initiation at referral sites, such as accommodating walk-in appointments and reducing patient paperwork burden.

Integrating MOUD into SSP programming also appears promising. Our review identified numerous SSP-based buprenorphine treatment programs that have published clinical data. Modeling suggests that widespread implementation of buprenorphine treatment at SSPs could dramatically decrease overdoses and OUD-related costs [70]; however, rigorous clinical trials of SSP-based buprenorphine treatment have not been conducted. We were also unable to identify studies that evaluated other evidence-based SUD treatments onsite at SSPs, including behavioral interventions (i.e. contingency management) or injectable buprenorphine; however, SSP participants’ interest in SUD and mental health treatment suggests demand for integrated services. SSP participants should be involved in the design and implementation of integrated services to help prevent medicalization of SSPs and ensure that the harm reduction ethos is preserved. Integrated services could include physically separate treatment and harm reduction spaces, close partnerships with nearby health centers with SSP staff acting as patient navigators, and/or telehealth services delivered onsite at the SSP [50, 53, 71]. Telehealth, which does not require exam rooms or clinic spaces, may be an important, low-barrier way for more SSPs to offer onsite MOUD [52]. The next step in this research could include effectiveness studies with different models of SSP-based SUD treatment and implementation studies to understand how to build and maintain programs in different geographic settings (i.e. rural vs. urban).

A strength of this study is that, to our knowledge, this is the first review to synthesize the literature on onsite SUD treatment at SSPs. We used a rigorous approach to identify studies and select them for inclusion, and we were able to answer several interrelated questions. There are also some limitations. In our review of descriptive studies, there were not sufficient data to assess whether some participant characteristics (e.g., housing status or current injection drug use) were associated with treatment utilization/entry. We describe associations that were significant across multiple studies, but study methods do not allow us to draw firm conclusions regarding causality. We limited our search to PubMed as we decided that the most important papers on medical interventions would be found using this database. There were only a few randomized controlled trials, which would provide the strongest evidence for the efficacy of interventions. Finally, we are unable to rigorously compare outcomes between countries because we only found one RCT conducted outside the United States.

In conclusion, more than 30 years of research has unequivocally demonstrated that SSPs decrease community HIV transmission, but less research has focused on how SSPs facilitate SUD treatment entry. Our review identified evidence-based practices to link SSP participants to SUD treatment, interest in onsite SUD treatment at SSPs, and promising models of onsite buprenorphine treatment, including opportunities to expand services using telehealth. Though efforts will also be necessary to improve the availability, accessibility, affordability, and acceptability of SUD treatment to SSP participants within the conventional healthcare system, our review demonstrates the important role that SSPs can play in reaching out-of-treatment PWUD. For too long, harm reduction and SUD treatment have been viewed in opposition, but the studies reviewed here provide models for successful collaboration.

## Data Availability

The datasets used and/or analyzed during the current study are available from the corresponding author on reasonable request.

## Appendix A

Final Search Criteria

(“Needle Exchange Program” OR “Needle Exchange Programs” OR “Needle Exchange Programme” OR “Needle Exchange Programmes” OR “Needle Exchange Center” OR “Needle Exchange Centers” OR “Needle Exchange Centre” OR “Needle Exchange Centres” OR “Needle Exchange Site” OR “Needle Exchange Sites” OR “Needle Exchange Facility” OR “Needle Exchange Facilities” OR “Needle Exchange Organization” OR “Needle Service Program” OR “Needle Service Programs” OR “Needle Service Programme” OR “Needle Service Programmes” OR “Needle Service Center” OR “Needle Service Centers” OR “Needle Service Centre” OR “Needle Service Centres” OR “Needle Service Site” OR “Needle Service Sites” OR “Needle Service Facility” OR “Needle Service Facilities” OR “Needle Service Organization” OR “Syringe Exchange Program” OR “Syringe Exchange Programs” OR “Syringe Exchange Programme” OR “Syringe Exchange Programmes” OR “Syringe Exchange Center” OR “Syringe Exchange Centers” OR “Syringe Exchange Centre” OR “Syringe Exchange Centres” OR “Syringe Exchange Site” OR “Syringe Exchange Sites” OR “Syringe Exchange Facility” OR “Syringe Exchange Facilities” OR “Syringe Exchange Organization” OR “Syringe Service Program” OR “Syringe Service Programs” OR “Syringe Service Programme” OR “Syringe Service Programmes” OR “Syringe Service Center” OR “Syringe Service Centers” OR “Syringe Service Centre” OR “Syringe Service Centres” OR “Syringe Service Site” OR “Syringe Service Sites” OR “Syringe Service Facility” OR “Syringe Service Facilities” OR “Syringe Service Organization” OR “Harm Reduction Program” OR “Harm Reduction Programs” OR “Harm Reduction Programme” OR “Harm Reduction Programmes” OR “Harm Reduction Center” OR “Harm Reduction Centers” OR “Harm Reduction Centre” OR “Harm Reduction Centres” OR “Harm Reduction Site” OR “Harm Reduction Sites” OR “Harm Reduction Facility” OR “Harm Reduction Facilities” OR “Harm Reduction Organization”) AND (referral OR linkage OR “medical care” OR detox* OR rehab* OR “intensive outpatient” OR methadone OR buprenorphine OR naltrexone OR “substance use disorder treatment” OR “primary care” OR “pre-exposure prophylaxis” OR “post-exposure prophylaxis” OR “antiretroviral therapy” OR “HCV” OR “HIV” OR "medications for opioid use disorder” OR “medication assisted treatment” OR “opioid treatment program” OR “methadone maintenance treatment program” OR “opioid agonist treatment” OR “opioid substitution therapy”).

## Appendix B

Data Extraction template

## Appendix C

List of all studies by category

Descriptive studies of treatment utilization/entry:

1. *Pilot study to enhance HIV care using needle exchange-based health services for out-of-treatment injecting drug users.* Altice, 2003.
2. *Help-seeking and referrals in a needle exchange: a comprehensive service to injecting drug users*. Carvell, 1990.
3. *A peer-led mobile outreach program and increased utilization of detoxification and residential drug treatment among female sex workers who use drugs in a Canadian setting*. Deering, 2011.
4. *A high proportion of users of low-threshold facilities with needle exchange programmes in Switzerland are currently on methadone treatment: implications for new approaches in harm reduction and care.* Gervasoni, 2012.
5. *Reduced injection frequency and increased entry and retention in drug treatment associated with needle-exchange participation in Seattle drug injectors*. Hagan, 2000.
6. *Can syringe exchange serve as a conduit to substance abuse treatment?* Heimer, 1998.
7. *Development of an enhanced needle and syringe programme: the First Step programme pilot.* Hudoba, 2004.
8. *Psychiatric distress, risk behavior, and treatment enrollment among syringe exchange participants*. Kidorf, 2010.
9. *Needle exchange program utilization and entry into drug user treatment: is there a long-term connection in Baltimore, Maryland?* Latkin, 2006.
10. *Bridge to services: drug injectors' awareness and utilization of drug user treatment and social service referrals, medical care, and HIV testing provided by needle exchange programs.* Porter, 2002.
11. *Drug user treatment referrals and entry among participants of a needle exchange program.* Riley, 2002.
12. *Needle-exchange attendance and health care utilization promote entry into detoxification*. Strathdee, 1999.
13. *Motivation to Change and Treatment Participation Among Syringe Service Program Utilizers in Rural Kentucky*. Surratt, 2020.

Linkage intervention studies

1. *Malmo Treatment Referral and Intervention Study (MATRIS)– effective referral from syringe exchange to treatment for heroin dependence: a pilot randomized controlled trial.* Bråbäck, 2016.
2. *The effect of a case management intervention on drug treatment entry among treatment-seeking injection drug users with and without comorbid antisocial personality disorder*. Havens, 2007.
3. *Challenges in motivating treatment enrollment in community syringe exchange participants.* Kidorf, 2005.
4. *Improving treatment enrollment and re-enrollment rates of syringe exchangers: 12-month outcomes*. Kidorf, 2009.
5. *A treatment reengagement intervention for syringe exchangers.* Kidorf, 2011.
6. *Improving substance abuse treatment enrollment in community syringe exchangers.* Kidorf, 2012.
7. *Facilitating entry into drug treatment among injection drug users referred from a needle exchange program: Results from a community-based behavioral intervention trial.* Strathdee, 2006.
8. *Development and evaluation of a community-based buprenorphine treatment intervention.* Fox, 2017*.*

Post-linkage outcomes

1. *Predictors of opiate agonist treatment retention among injection drug users referred from a needle exchange program.* Havens, 2009.
2. *Malmo Treatment Referral and Intervention Study-High 12-Month Retention Rates in Patients Referred from Syringe Exchange to Methadone or Buprenorphine/Naloxone Treatment.* Bråbäck, 2017.
3. *Treatment initiation strategies for syringe exchange referrals to methadone maintenance: A randomized clinical trial.* Kidorf, 2018.
4. *Reducing Risky Drug Use Behaviors by Enrolling Syringe Exchange Registrants in Methadone Maintenance.* Kidorf, 2021.
5. *Sexual-risk reduction following the referral of syringe exchange registrants to methadone maintenance: Impact of gender and drug use.* Kidorf, 2021.
6. *Benefits of concurrent syringe exchange and substance abuse treatment participation.* Kidorf, 2011.
7. *Feasibility of referring drug users from a needle exchange program into an addiction treatment program: experience with a mobile treatment van and LAAM maintenance.* Kuo, 2003.
8. *Drug abuse treatment success among needle exchange participants.* Brooner, 1998..
9. *A comparison of 1-year substance abuse treatment outcomes in community syringe exchange participants versus other referrals*. Neufeld, 2008.

Onsite treatment

1. *Description and outcomes of a buprenorphine maintenance treatment program integrated within Prevention Point Philadelphia, an urban syringe exchange program.* Bachhuber, 2018.
2. *Interest in long-acting injectable buprenorphine among syringe services program participants.* Epstein, 2021.
3. *Illicit buprenorphine use, interest in and access to buprenorphine treatment among syringe exchange participants*. Fox, 2015.
4. *Harm Reduction Agencies as a Potential Site for Buprenorphine Treatment.* Fox, 2015.
5. *“We’ll be able to take care of ourselves”—A qualitative study of client attitudes toward implementing buprenorphine treatment at syringe services programs.* Frost, 2021.
6. *Initiation of Low-threshold Buprenorphine in Nontreatment Seeking Patients With Opioid Use Disorder Engaged in Hepatitis C Treatment.* Hill, 2022.
7. *Engaging an unstably housed population with low-barrier buprenorphine treatment at a syringe services program: Lessons learned from Seattle, Washington.* Hood, 2020.
8. *Low-threshold Buprenorphine Treatment in a Syringe Services Program: Program Description and Outcomes.* Jakubowski, 2021.
9. *Implementation of Buprenorphine Services in NYC Syringe Services Programs: a qualitative process evaluation*. Jakubowski, 2022.
10. *Buprenorphine implementation at syringe service programs following waiver of the Ryan Haight Act in the United States.* Lambdin, 2022.
11. *Improving equity and access to buprenorphine treatment through telemedicine at syringe services programs.* Lambdin, 2022.
12. *Mobile low-threshold buprenorphine integrated with infectious disease services.* Rosecrans, 2022.
13. *Concurrent Initiation of Hepatitis C and Opioid Use Disorder Treatment in People Who Inject Drugs.* Rosenthal, 2020.
14. *Opioid maintenance treatment as a harm reduction tool for opioid-dependent individuals in New York City: the need to expand access to buprenorphine/naloxone in marginalized populations*. Stancliff, 2012.

Review Articles Spanning Categories

1. *Expanding the public health benefits of syringe exchange programs*. Kidorf, 2008.
2. *Integrated Models of Care for Individuals with Opioid Use Disorder: How Do We Prevent HIV and HCV?* Rich, 2018.
3. *Scoping out the literature on mobile needle and syringe programs-review of service delivery and client characteristics, operation, utilization, referrals, and impact.* Strike, 2018.

National surveys describing health services (including SUD treatment) provided by SSPs

1. *Doing harm reduction better: syringe exchange in the United States.* Des Jarlais, 2009.
2. *Harm reduction and health services provided by syringe services programs in 2019 and subsequent impact of COVID-19 on services in 2020.* Behrends, 2022

Participants’ preferences regarding SUD treatment

1. *Designing an “Ideal” Substance Use Disorder Treatment Center: Perspectives of People Who Have Utilized Medications for Opioid Use Disorder.* Andraka-Christou, 2021.
2. *Consumer attitudes about opioid addiction treatment: a focus group study in New York City.* Sohler, 2013.

## Abbreviations

ASI: Addiction severity index
HCV: Hepatitis C virus
LAAM: Levo-alpha acetyl methadol
MOUD: Medications for OUD
MRC: Motivational referral condition
OUD: Opioid use disorder
PrEP: Pre-exposure prophylaxis
PWID: People who inject drugs
PWUD: People who use drugs
SBCM: Strength-based case management
SSP: Syringe services program
SUD: Substance use disorders
UDT: Urine drug testing
RCT: Randomized controlled trial
